# Influence of different drug delivery methods for Endostar combined with a gemcitabine/cisplatin regimen in locally advanced or metastatic lung squamous cell carcinoma: A retrospective observational study

**DOI:** 10.1097/MD.0000000000011822

**Published:** 2018-08-10

**Authors:** Difei Yao, Hong Shen, Jianjin Huang, Ying Yuan, Haibin Dai

**Affiliations:** a Department of Pharmacy; b Department of Oncology, Second Affiliated Hospital, Zhejiang University School of Medicine, Hangzhou, China.

**Keywords:** continuous administration, Endostar, recombinant human endostatin, squamous cell carcinoma

## Abstract

Continuous endovenous administration of Endostar (CE) gradually replaced drip intravenous administration of Endostar (DE) in lung squamous cell carcinoma (SCC) treatment presently, but the efficacy and safety of CE and DE which is better in advanced lung SCC are yet unclear. To evaluate the feasibility of CE as an alternative to DE with gemcitabine/cisplatin (GP) chemotherapy. Data were collected from patients admitted with locally advanced or metastatic lung SCC from January 2011 to April 2015, including the patients’ characteristics, the therapeutic regimen, the treatment effectiveness, and toxicity. There are 71 patients with pathologically confirmed lung SCC retrospectively assigned to a treatment (CE) group of 48 patients and a control (DE) group of 23 patients. The response of each tumor to the therapy was assessed every 2 cycles by a chest and upper abdomen computed tomography for the comparison of curative effects and adverse reactions. Compared with the DE group, the response rate and disease control rate were noninferior in the CE group. The median progression-free survival and overall survival in the CE and DE groups were no significantly difference (5.5 vs 5.5 months, *P* = .141; 22.9 vs 14.3 months, *P* = .053). Increased progression-free survival (PFS) for patients in CE group was observed across 3 subgroups analyzed. There was a 35.7% reduction in the total dose of Endostar per cycle in the CE group compared with that in the DE group. Thus, in combination with GP chemotherapy, CE could be a suitable alternative to DE in locally advanced or metastatic SCC patients, resulting in less hemoptysis, less treatment time, and lower costs.

## 1 Introduction

Lung cancer, the leading cause of cancer deaths worldwide, accounted for 1.59 million deaths in 2012.^[1]^ In China, lung cancer replaced liver cancer as the primary cause of death among cancer patients since 2008.^[2]^ Non-small-cell lung cancer (NSCLC) accounts for almost 85% of all lung cancer cases. Among lung cancer patients, approximately 27% are diagnosed with squamous cell carcinoma (SCC).^[3]^

Endostar (YH-16), approved by China's State Food and Drug Administration for the treatment of NSCLC in 2005, is a novel recombinant human endostatin. In 2005, a phase III study in patients with advanced NSCLC showed synergic activity of Endostar in combination with vinorelbine plus cisplatin.^[4]^ Since then, other clinical studies have also been conducted to assess Endostar's effectiveness in treating lung SCC.^[5,6]^ Compared with other angiogenesis inhibitors, neither severe pulmonary hemorrhage nor mortality risk increase was reported in SQCLC patients treated with Endostar.^[7]^ The safety profile of Endostar is acceptable so far, but the clinical benefit is still limited using the traditional drip endovenous administration method.

Based on the Folkman theory^[8]^ and preclinical data,^[9]^ since 2012, continuous endovenous administration of Endostar (CE) gradually replaced drip intravenous administration of Endostar (DE) in lung SCC^[10,11]^ and other cancer treatment.

Although compared with gemcitabine plus cisplatin (GP) chemotherapy alone, the CE combined with GP chemotherapy improved progression-free survival (PFS) in local advanced or metastatic lung SCC in our former study.^[10]^ It did not elucidate if the drug delivery method of Endostar has an effect on the efficacy of Endostar in patients with this disease. To our knowledge, the efficacy and toxicity of CE and DE with GP chemotherapy in local advanced or metastatic lung SCC have not been head to head compared yet.

In this study, a comparison of the efficacy of and adverse reactions toward the CE combined with GP chemotherapy and DE combined with GP chemotherapy in advanced lung SCC patients was retrospectively investigated.

## 2 Materials and methods

### 2.1 Patients

This retrospective comparative study was conducted at the 2nd Affiliated Hospital, Zhejiang University School of Medicine from May 2015 to January 2018, with the approval of the Institutional Review Board of the 2nd Affiliated Hospital, Zhejiang University School of Medicine. There was no outpatient received intravenous chemotherapy in our hospital, thus this study was conducted among hospitalized patients. We retrospectively reviewed data from 97 patients admitted with locally advanced or metastatic lung SCC from January 2011 to April 2015 who used GP plus Endostar. We excluded 18 potential resection patients who used GP plus Endostar as neoadjuvant therapy, 5 patients used GP once for progression of disease, 3 patients used Endostar once for economic reason. All of the patients were histologically or cytologically confirmed to have unrespectable stage IIIB or IV SCC with an Eastern Cooperative Oncology Group performance status of 0 to1 and a life expectancy > 3 months. Seventy-one eligible lung SCC patients were included. Follow-up was made by telephone after hospitalization. All of the patients had at least 1 measurable tumor lesion. Patients were retrospectively assigned to a CE group and a DE group on the basis of different drug delivery methods for Endostar. Data were collected from 71 patients, including the patients’ characteristics, the therapeutic regimen, the treatment effectiveness, and toxicity. Due to the retrospective nature of the study, informed consent was waived.

### 2.2 Treatment

For all patients, the treatment cycle was 21 days. The GP chemotherapy was administered as follows: 1000 mg/m^2^ of gemcitabine on days 1 and 8 and 25 mg/m^2^ of cisplatin from day 1 to day 3. Patients in the CE group received 15 mg of Endostar (diluted in 250 mL of normal saline) daily using an automatic drug infusion pump (ZZB-II; Nantong Apon Medical Appliance Co, Ltd, Nantong, China) via a central line at the speed of 11 mL per hour from day 0 to 8 prior to the chemotherapy. For the DE group, 15 mg Endostar (diluted in 500 mL of normal saline) was administered by intravenous infusion over 4 hours from day 0 to day 13 before chemotherapy.

### 2.3 Assessment of the therapeutic response and adverse effects

Before and during the treatment, the results of physical examinations, complete blood counts, comprehensive blood chemistries, abdominal and chest computed tomography scans, brain magnetic resonance imaging scans, bone emission computed tomography (ECT), and positron emission tomography computed tomography (PET-CT) were recorded. According to the Response Evaluation Criteria in Solid Tumors 1.1 guidelines, the response of each tumor to the therapy was assessed every 2 cycles by a chest and upper abdomen computed tomography. The objective response rate (ORR), disease control rate (DCR), PFS, and overall survival (OS) were evaluated. Drug side effects were tracked according to the National Cancer Institute Common Terminology Criteria Adverse Events (NCI-CTCAE) version 4.0.

### 2.4 Statistical analysis

All of the categorical variables, objective response rates, and incidences of adverse effects were analyzed and compared between the 2 groups using the *χ*^2^ test or Fisher exact test, as appropriate. The distributions of the PFS and OS were estimated using the Kaplan–Meier method. The CE group and DE group were compared using the log-rank test. All *P* values were 2-sided, and values less than .05 were considered significant. All of the analyses were performed using SPSS 20.0 software (SPSS Inc, Chicago, IL).

## 3 Results

### 3.1 Baseline characteristics of the patients

Seventy-one patients with advanced lung SCC were included in this study. Their baseline characteristics are presented in detail in Table 1. All of the patients’ characteristics were comparable. The median age of the patients was 63 in both groups. Five of the 48 (97.9%) patients in the CE group and 22 of the 23 (95.7%) patients in the DE group were men. Forty-four (91.7%) and 21 (91.3%) of the patients included in the CE and DE groups, respectively, were not current and had never been smokers. The grade according to the Eastern Cooperative Oncology Group (ECOG) performance status (PS) of 48 patients in the CE group and 22 patients in the DE group was 0 to 1. None of the patients in the CE group and 1 patient in the DE group had an ECOG PS of 2. There were 78.3% and 68.8% stage IV patients in the DE and CE groups, respectively.

**Table 1:**
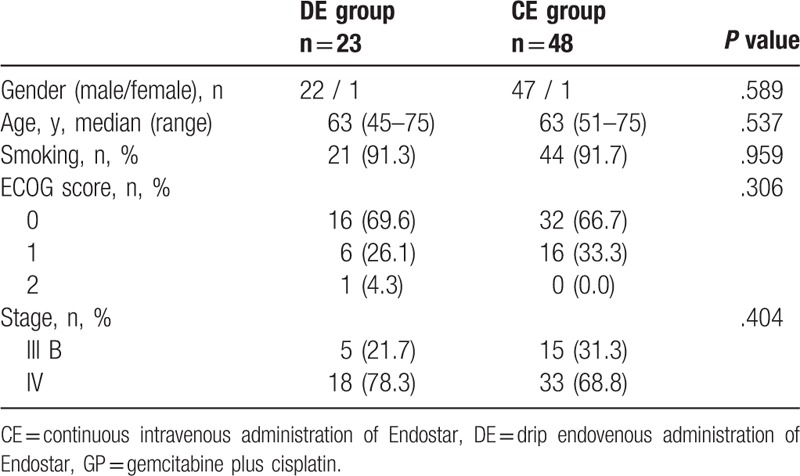
Baseline characteristics of the locally advanced or metastatic lung squamous cell carcinoma patients.

### 3.2 Efficacy

**Figure 1:**
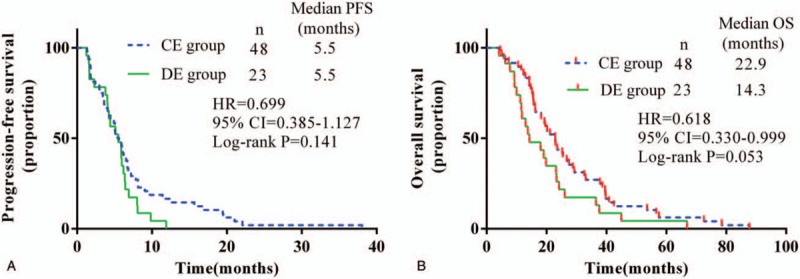
Kaplan–Meier estimated survival for locally advanced or metastatic lung squamous cell carcinoma patients treated with different drug delivery methods of Endostar plus a gemcitabine/cisplatin regimen. (A) Progression-free survival and (B) overall survival. CE = continuous intravenous administration of Endostar, CI = confidence interval, DE = drip intravenous administration of Endostar, HR = hazard ratio, OS = overall survival, PFS = progression-free survival.

The median follow-up was 16.9 months in the CE group and 10.7 months in the DE group. None of these patients achieved complete remission (CR) by the cut-off time (January 18, 2018). The number of partial remission (PR) cases was 21 and 12 in the CE and DE groups, respectively. Twenty cases of stable disease (SD) were observed in the CE group and 7 were found in the DE group. Seven cases in the CE group and 2 cases in the DE group were evaluated as progressive disease (PD). The response rate (RR) in the CE group was 45.8%, compared with 47.8% in the DE group (*P* = .875). The DCR was 85.4% in the CE group and 90.5% in the DE group (*P* = .760). The detailed efficacy data are shown in Table 2. Compared with the DE group, the median PFS and OS were noninferior in the CE group (5.5 vs 5.5 months, *P* = .141; 22.9 vs 14.3 months, *P* = .053) (Fig. 1).

**Table 2:**
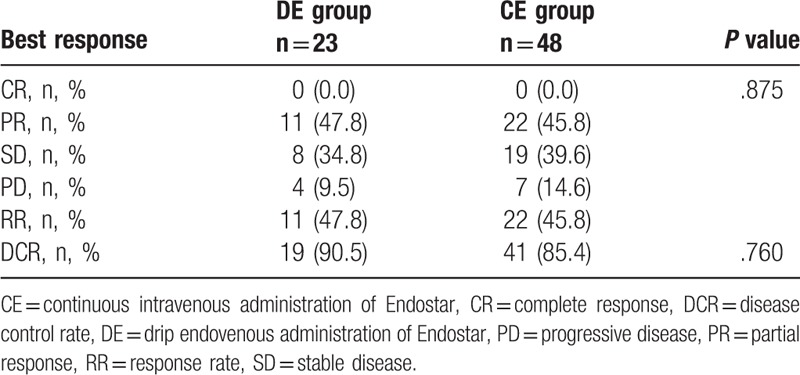
Response and control rates for different drug delivery methods of Endostar combined with a GP regimen in locally advanced or metastatic lung squamous cell carcinoma.

**Figure 2:**
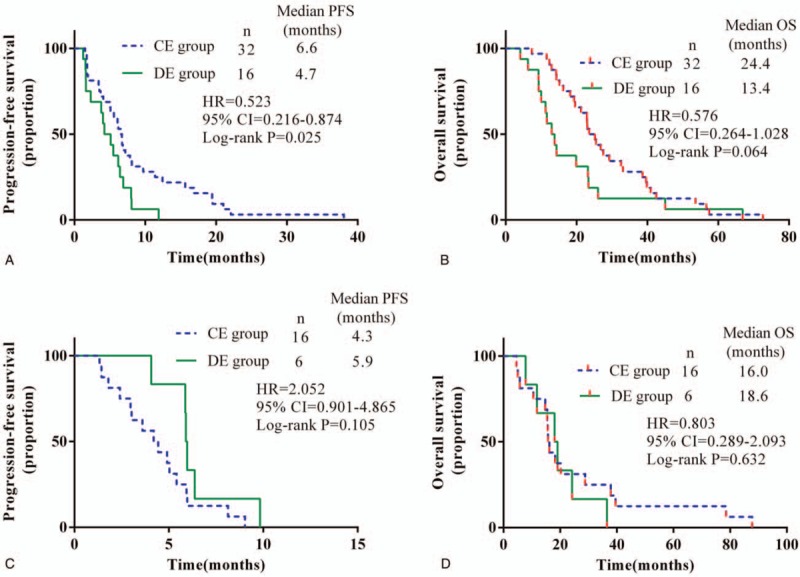
Kaplan–Meier estimated progression-free survival (PFS) and overall survival (OS) for locally advanced or metastatic lung squamous cell carcinoma patients treated with different drug delivery methods of Endostar plus a gemcitabine/cisplatin regimen. (A) PFS and (B) OS in patients with ECOG PS score 0; (C) PFS and (D) OS in patients with ECOG PS score 1. CE = continuous intravenous administration of Endostar, CI = confidence interval, DE = drip endovenous administration of Endostar, HR = hazard ratio.

Unstratified exploratory subgroup analyses of both the PFS and OS were performed. In patients with an ECOG PS score of 0 (n = 48), PFS favored the CE group (n = 32, HR = 0.523, *P* = .025), as did time to alternative Endostar delivery method (6.6 months CE group, 4.7 months DE group, *P* = .025). Although the median OS is longer in the patients in the CE group, there was no statistical difference between these 2 groups (24.4 months CE group, 13.4 months DE group, *P* = .064) (Fig. 2, A and B). In another 23 lung SCC patients with an ECOG PS score of 1, the grouping had no effect on the PFS or OS (Fig. 2, C and D).

**Figure 3:**
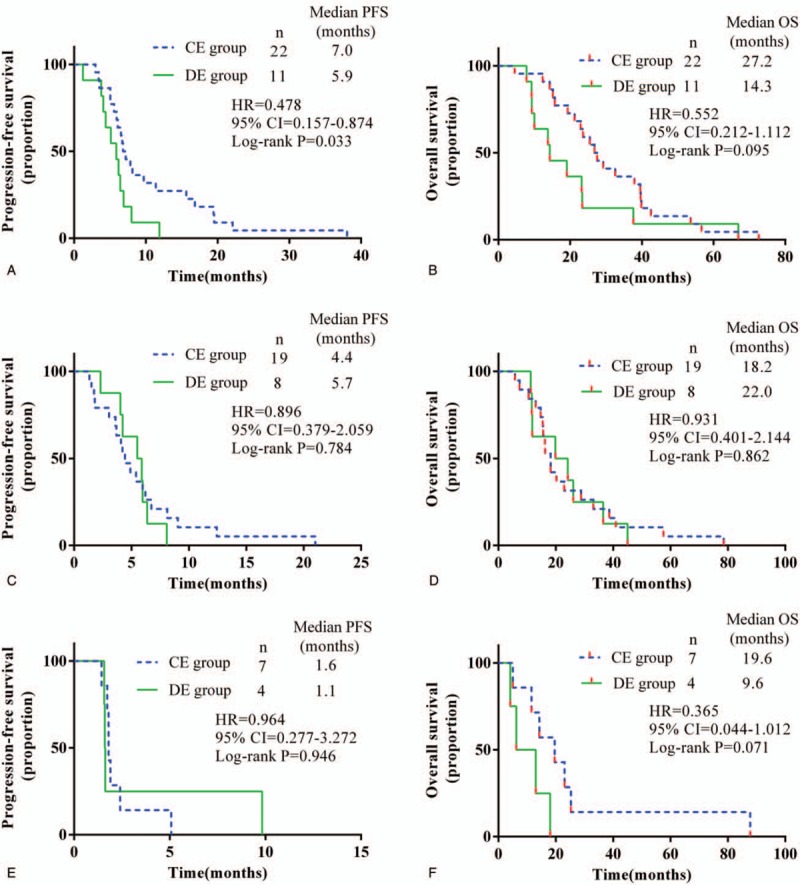
Kaplan–Meier estimated progression-free survival (PFS) and overall survival (OS) for locally advanced or metastatic lung squamous cell carcinoma patients treated with different drug delivery methods of Endostar plus a gemcitabine/cisplatin regimen. (A) PFS and (B) OS in patients with partial response; (C) PFS and (D) OS in patients with stable disease; (E) PFS and (F) OS in patients with progressive disease. CE = continuous intravenous administration of Endostar, CI = confidence interval, DE = drip endovenous administration of Endostar, HR = hazard ratio.

In patients achieved PR in this study (n = 33), PFS was significantly longer with CE than with DE (7.0 vs 5.9 months, *P* = .033) (Fig. 3A). The OS of these patients was not significantly different between groups (27.2 months CE group, 14.3 months DE group, *P* = .095) (Fig. 3B). In patients achieved SD or PD in this study (n = 38), there was no difference of statistics in PFS or OS of patients in CE or DE groups (Fig. 3, C–E).

**Figure 4:**
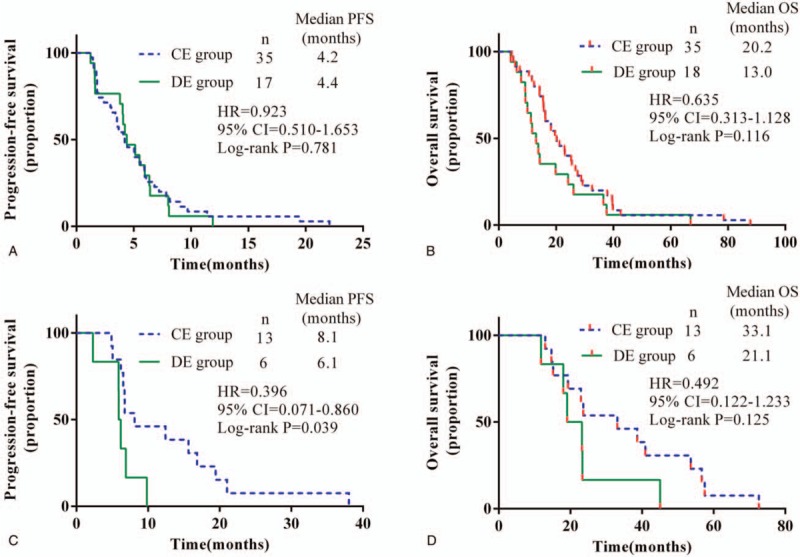
Kaplan–Meier estimated progression-free survival (PFS) and overall survival (OS) for locally advanced or metastatic lung squamous cell carcinoma patients treated with different drug delivery methods of Endostar plus a gemcitabine/cisplatin regimen. (A) PFS and (B) OS in patients treated with no more than 4 courses of Endostar; (C) PFS and (D) OS in patients treated with more than 4 courses of Endostar. CE = continuous intravenous administration of Endostar, CI = confidence interval, DE = drip endovenous administration of Endostar, HR = hazard ratio.

Among the patients treated with Endostar for less than or equal to 4 courses (n = 52), different drug delivery methods of Endostar did not have a significant influence on the PFS or OS in advanced lung SCC patients (Fig. 4, A and B). Among the patients who underwent Endostar treatment for more than 4 courses (n = 19), CE seemed to improve the PFS (Fig. 4C) but not the OS (Fig. 4D).

**Figure 5:**
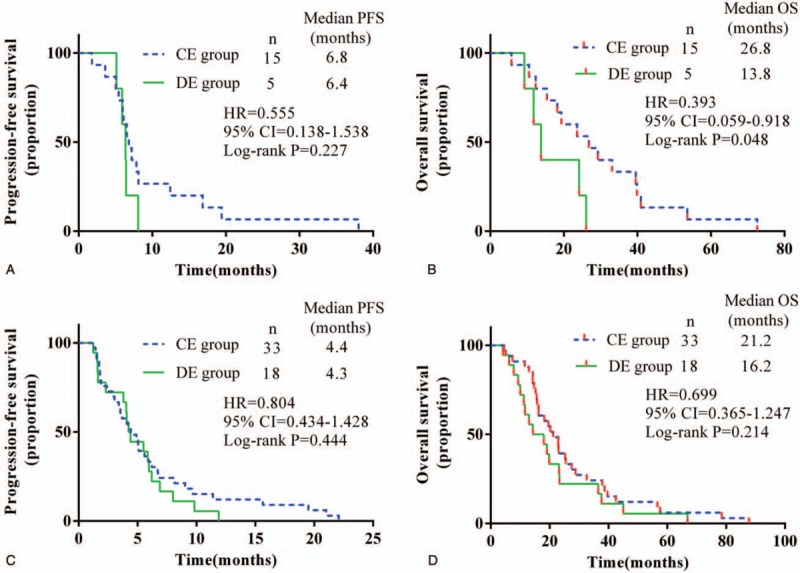
Kaplan–Meier estimated progression-free survival (PFS) and overall survival (OS) for locally advanced or metastatic lung squamous cell carcinoma (SCC) patients treated with different drug delivery methods of Endostar (more than 4 courses) plus gemcitabine/cisplatin regimen. (A) PFS and (B) OS in patients with lung SCC stage III; (C) PFS and (D) OS in patients treated with lung SCC stage IV. CE = continuous intravenous administration of Endostar, CI = confidence interval, DE = drip endovenous administration of Endostar, HR = hazard ratio.

In patients with stage III lung SCC of CE group (n = 15), CE seemed to improve the OS (26.8 months CE group, 13.8 months DE group, *P* = .048) (Fig. 5B) but not the PFS (Fig. 5A). In patients with metastatic disease, there is indifference in PFS or OS between the 2 groups (Fig. 5, C and D).

### 3.3 Toxicity

The patients were monitored for hematologic and nonhematologic toxicities from the treatment. The toxicity analysis was based on the NCI-CTCAE version 4.0. None of the patients died of adverse effects. The hematologic and nonhematologic toxicities are listed, respectively, in Tables 3 and 4, measured in grades of severity ranging from 0 to 5. No grade-five toxicity was reported in this study. Hemoptysis was the only adverse reaction that was significantly worse in the control group (*P* = .039). Neutropenia was the predominant hematologic toxic reaction in both the groups. In addition to nausea, increased alanine aminotransferase (ALT) or aspartate aminotransferase (AST) was the most common nonhematologic adverse reaction observed.

**Table 3:**
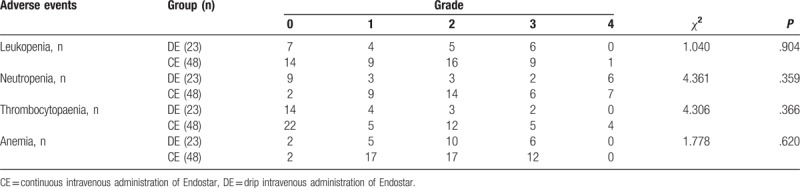
Treatment-related hematologic toxic reactions toward different drug delivery methods of Endostar combined with a GP regimen in locally advanced or metastatic lung squamous cell carcinoma.

**Table 4:**
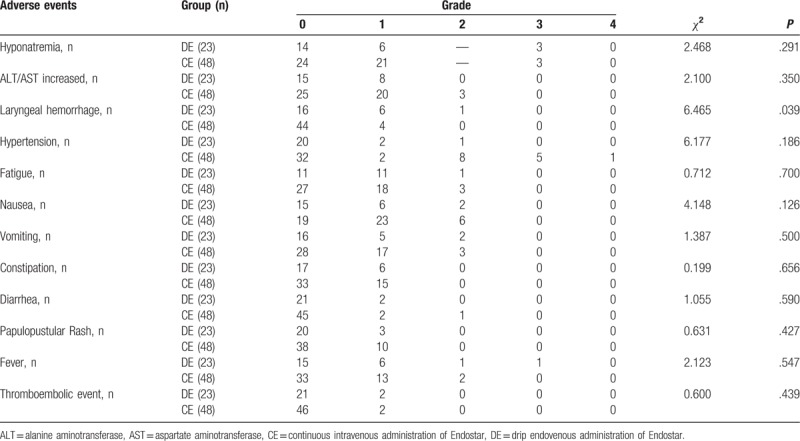
Treatment-related nonhematologic toxic reactions toward different drug delivery methods of Endostar combined with a GP regimen in locally advanced or metastatic lung squamous cell carcinoma.

## 4 Discussion

For lung SCC patients, GP is potentially better than other cisplatin-based chemotherapy regimens, but its benefit is still limited.^[12]^ Novel molecular targets specific to SCC patients are urgently needed.^[13]^ Although antiangiogenesis therapy and immunotherapy have been studied for lung SCC, due to safety concerns and little benefit to the OS,^[14]^ progress has been slow.

Endostatin, a 20-kD internal fragment of the carboxyl terminus of collagen XVIII, was first isolated in 1997.^[15]^ According to expanding concepts of angiogenesis, low doses of broader-spectrum angiogenesis inhibitors are more effective than higher doses and that continuous exposure to angiogenesis inhibitors is needed to treat cancer effectively.^[8]^

The half-life of endostatin in vivo is short.^[16]^ In healthy volunteers, Endostar has a terminal clearance half-life of approximately 10 hours after a single intravenous drip of 30 and 60 mg/m^2^ within 30 minutes (at a speed of 1 and 2 mg/m^2^/min, respectively) and of 120 and 210 mg/m^2^ within 120 minutes (at a speed of 1 and 1.75 mg/m^2^/min, respectively). Endostar showed linear pharmacokinetics within the dose range from 30 to 120 mg/m^2^ in these subjects. In cancer patients, the drug concentration time curve of Endostar after a daily intravenous drip for 2 hours over 28 days shows great differences between individuals. An increase in the administration time tends to an increase the minimum concentration.^[19]^ Based on preclinical data,^[17,18]^ the sustained delivery of Endostar may be advantageous.

In this study, we compared the efficacy of the CE to the DE both plus GP chemotherapy, in advanced SQCLC patients. These 2 groups showed no significant differences in PFS. Although the mOS in the CE group is longer compared with the DE group (22.9 months vs 14.3 months), the difference was not statistically significant either. For power calculation, we assumed an equal standard deviation in these 2 groups. With α=0.05, 2-tailed and a power of 80%, we needed 63 patients in the DE group and 130 patients in the CE group to achieve statistical differences. As a retrospective study and the almost clinical outage of DE, the sample number is limited in this research. Study with larger sample number could be performed to further verify our conclusions.

As Endostar is usually used with chemotherapy or radiotherapy as a synergistic treatment, few studies have reported adverse reactions to Endostar individually. In Phase I to III clinical trials, the incidence of toxicity and moderate and severe side effects was not significantly different between the DE group and the group without Endostar.^[4]^ Subsequently, the researchers published a safety profile reflecting these results.^[7,20]^ In other studies, cardiac toxicity seemed to occur occasionally in patients receiving Endostar treatment.^[21]^ In these studies, DE was administrated for 3 hours. Since sustained administration may augment the anticancer effect of Endostar, it is unclear whether this type of administration results in additional adverse reactions. In a preclinical safety study, 14 mg/kg of CE was injected intraperitoneally into healthy mice over 7 days. Additionally, 2 mg/kg of DE was injected using the same method for 7 days. At the end of the experiment, no detectable sign of injury was found in the myocardial, lung, and kidney tissues in either group. The cell fraction of CD11b-, CD146+, and CD105- vascular endothelial cells in the peripheral blood was higher in the CE group than in the DE group. The author suggested that CE may promote injury to the endothelium.^[9]^

Unlike the results of the preclinical study, in our study and in a clinical study, there was no obvious cardiac toxicity observed in patients who received 15 mg of Endostar continuously.^[20]^ In our study, 4 patients had ST-segment and T-wave changes (2 in each group, 4.2% in the CE group and 8.7% in the DE group, *P* = .168). After suspending Endostar and chemotherapy, these changes disappeared, and treatment was subsequently continued. In this study, the CE reduced the risk of hemoptysis. In the CE group, 4 patients (8.3%) developed hemoptysis; however, in the DE group, 7 patients (30.4%) developed hemoptysis. This finding suggests that continuous administration may be a safer drug-delivery route for Endostar.

Stratification exploratory subgroup analyses suggested that patients with ECOG PS score 0, PR as best treatment effect or treated with more than 4 courses of Endostar may achieve longer PFS than patients with the same condition in the DE group. In stage III lung SCC patients, we observed an obviously longer OS in patients of CE group than DE group. But since there was not same effect on PFS, we consider that should be more data to draw a conclusion. We assume that DE plus GP is more suitable for advanced lung SCC patients in good physical condition and sensitive to Endostar plus GP treatment. On the other hand, if an advanced lung SCC patient who ECOG PS score is more than 0 or fail to achieve PR or CR in his treatment, the DE might be a better choice.

In addition, as the disease burden of lung cancer has obviously increased over the past decades,^[22]^ it is important to identify more economic treatment methods as well. Compared with the traditional DE, continuous administration can achieve a 35.7% reduction in the total dose of Endostar per cycle. According to the price of Endostar in 2015, 1 patient could save CNY 4307 per cycle on Endostar. Because the CE is not inferior to DE in advanced SQCLC patients, the former may be a more economical choice.

In combination with GP chemotherapy, the CE was not inferior to the DE in locally advanced or metastatic SQCLC patients, exhibiting a similar adverse reaction profile while being more economical. This study was a single-center, nonrandomized, retrospective research. A subsequent large-sample prospective clinical trial is needed to confirm the efficacy and safety of the continuous intravenous administration of Endostar plus chemotherapy in advanced lung SCC patients.

## Author contributions

**Data curation:** Difei Yao, Hong Shen.

**Formal analysis:** Difei Yao, Hong Shen.

**Funding acquisition:** Difei Yao.

**Investigation:** Difei Yao.

**Methodology:** Jianjin Huang.

**Project administration:** Ying Yuan, Haibin Dai.

**Resources:** Jianjin Huang.

**Software:** Hong Shen.

**Supervision:** Jianjin Huang, Haibin Dai.

**Writing – original draft:** Difei Yao.

**Writing – review & editing:** Ying Yuan, Haibin Dai.

Haibin Dai (ORCID: 0000-0002-5768-2714)
